# Spinel-Based ZnAl_2_O_4_: 0.5%Cr^3+^ Red Phosphor Ceramics for WLED

**DOI:** 10.3390/ma17071610

**Published:** 2024-04-01

**Authors:** Wenchao Ji, Xueke Xu, Ming Qiang, Aihuan Dun

**Affiliations:** Shanghai Institute of Optics and Fine Mechanics, Chinese Academy of Sciences, No. 899 Huiwang East Road, Shanghai 201800, China

**Keywords:** phosphor ceramic, WLED, spinel, Cr^3+^

## Abstract

To address the issue of the lack of red light in traditional Ce^3+^: YAG-encapsulated blue LED white light systems, we utilized spark plasma sintering (SPS) to prepare spinel-based Cr^3+^-doped red phosphor ceramics. Through phase and spectral analysis, the SPS-sintered ZnAl_2_O_4_: 0.5%Cr^3+^ phosphor ceramic exhibits good density, and Cr^3+^ is incorporated into [AlO_6_] octahedra as a red emitting center. We analyzed the reasons behind the narrow-band emission and millisecond-level lifetime of ZAO: 0.5%Cr^3+^, attributing it to the four-quadrupole interaction mechanism as determined through concentration quenching modeling. Additionally, we evaluated the thermal conductivity and thermal quenching performance of the ceramic. The weak electron-phonon coupling (EPC) effects and emission from antisite defects at 699 nm provide positive assistance in thermal quenching. At a high temperature of 150 °C, the thermal conductivity reaches up to 14 W·m^−1^·K^−1^, and the 687 nm PL intensity is maintained at around 70% of room temperature. Furthermore, the internal quantum efficiency (IQE) of ZAO: 0.5%Cr^3+^ phosphor ceramic can reach 78%. When encapsulated with Ce^3+^: YAG for a 450 nm blue LED, it compensates for the lack of red light, adjusts the color temperature, and improves the color rendering index (R9). This provides valuable insights for the study of white light emitting diodes (WLEDs).

## 1. Introduction

Over the past two decades, progress in semiconductor light source technology has led to a substantial transformation in the field of illumination. White light emitting diodes (WLEDs), known for their high efficiency, environmental friendliness, compact size, and extended lifespan, have increasingly replaced traditional electric light sources such as incandescent bulbs, fluorescent lamps, and high-intensity discharge lamps [1,2,3]. Currently, solid-state light sources for white lighting are mainly based on phosphor-converted white LEDs (pc-WLEDs). Furthermore, many researchers, such as the US GE Research Institute and individuals like Kurtin [4,5], are dedicated to developing phosphor materials for WLEDs. Among the elements of pc-WLEDs, rare earth/transition metal-ion-activated inorganic phosphors, selected for their high efficiency, suitable emission colors, and good physicochemical stability, have become crucial materials for achieving WLEDs with excellent color and high color rendering index (CRI). 

In 1995, Shuji Nakamura of Nichia Chemical Industries invented the phosphor-converted white LED system, which first introduced yellow emitting yttrium aluminum garnet (YAG: Ce^3+^) phosphor coated on a blue LED to the market [6,7,8]. However, the white light obtained using this method lacks red components, limiting its application in high-end indoor lighting [9,10]. Therefore, there is a need to increase the red phosphor to improve the color rendering index and adjust the color temperature. To date, pc-WLEDs remain the most mainstream solution for achieving WLED illumination sources due to their low cost, high reliability, and mature technology. Different fluorescent powders in various red emission bands compensate for the improvement of the color rendering index (CRI) in different application scenarios based on the varying sensitivity of the human eye to red light waves. For instance, WLEDs used in high-demand settings like theater lighting require a CRI of 95, while WLED lighting in conventional places typically demands a CRI of around 80. Currently, commercially available red phosphor (Ca, Sr) AlSiN_3_: Eu^2+^ with Eu elements is expensive, and the nitride preparation process is complex [11,12]. Therefore, there is an urgent need to develop new luminescent materials with good performance that are simultaneously low cost and have a simple preparation process.

In this context, chromium (Cr^3+^) ions, as transition metal ions with abundant reserves and the potential to serve as red emitting centers, have garnered widespread attention as activators for LED phosphor materials [13,14,15,16,17]. Another major issue faced by pc-WLEDs is the poor thermal conductivity of the phosphor film due to the current packaging method, where transparent silicone gel and phosphors are mixed and encapsulated on LEDs [18]. In response to this challenge, high thermal conductivity fluorescent ceramics have emerged, especially suitable for high-power/high-brightness excitation [19,20,21].

Among the various matrices of phosphor ceramics, spinel ceramics are a relatively mature transparent technology [22,23,24]. Currently, magnesium aluminum spinel is the most widely researched and used. In comparison, zinc aluminum spinel has better acid and alkali resistance and higher thermal conductivity [25,26,27,28,29], and its [AlO_6_] octahedral coordination environment matches well with Cr^3+^ ions. It holds promise for the preparation of fluorescent ceramics for high-power/high-brightness WLEDs. We utilized spark plasma sintering (SPS) to prepare ZnAl_2_O_4_: xCr^3+^ (x = 0.3%, 0.4%, 0.5%, 0.6%, and 0.7%) phosphor ceramics. We conducted comprehensive characterization and analysis of their phase, luminescent properties, thermal quenching, etc. This was done to validate their potential application value in compensating for red light in traditional white light sources. 

## 2. Experimental Section

### 2.1. Materials and Synthesis

ZnAl_2_O_4_: xCr^3+^ (ZAO: xCr^3+^, x = 0.3%, 0.4%, 0.5%, 0.6%, and 0.7%) phosphor ceramics were synthesized using spark plasma sintering (SPS). Commercially raw materials, including ZnO (99.999%), Al_2_O_3_ (99.999%), Cr_2_O_3_ (99.999%), and LiF (99.999%), were used without additional purification. Precise amounts of the raw materials were measured according to specified ratios and thoroughly mixed with zirconia balls through ball milling. The resulting mixture underwent washing, and after drying in a 100 °C oven for 10 h, dry powders were obtained. The mixed powders were obtained after three rounds of screening with a 100-mesh sieve. To eliminate organic impurities, the obtained powders underwent sintering in a muffle furnace at 800 °C for 5 h. Subsequently, 2 g of the powder was weighed and placed into a graphite mold within the SPS for the final ceramic sintering. The sintering process was conducted at 1260 °C for 15 min. Finally, the sintered phosphor ceramics were subjected to double-sided polishing for testing.

### 2.2. Characterization

X-ray diffraction (Empyrean X’pert 3, Almelo, The Netherlands) within the 2θ range from 20° to 80° with Cu Kα irradiation (λ = 0.15418 nm) was used to examine the crystal phases of the phosphor ceramics. Photoluminescence excitation (PLE) spectra, temperature-dependent PL spectra (30 to 240 °C), quantum yield (QY), and PL decay curves were acquired by a fluorescence spectrophotometer (Edinburgh Instruments FLS1000, Livingston, UK). Diffuse reflection spectra (DRS) and transmittance spectra were carried out using an ultraviolet-visible-near-infrared spectrophotometer (Shimadzu SolidSpec-3700i/3700i DUV, Kyoto, Japan). Morphological features were investigated using a field-emission scanning electron microscope (SEM, Regulus 8100, Tokyo, Japan). Luminescence spectra of the phosphor-converted LED (pc-LED) assemblies were measured and calculated using an LED opto-electrical analyzer (Everfine ATA-500, Hangzhou, China). Additionally, the thermal conductivity of the ZnAl_2_O_4_: 0.5%Cr^3+^ phosphor ceramic was determined using the flash laser method (Netzch LFA-457, Serb, Germany).

## 3. Results and Discussion

**Figure 1:**
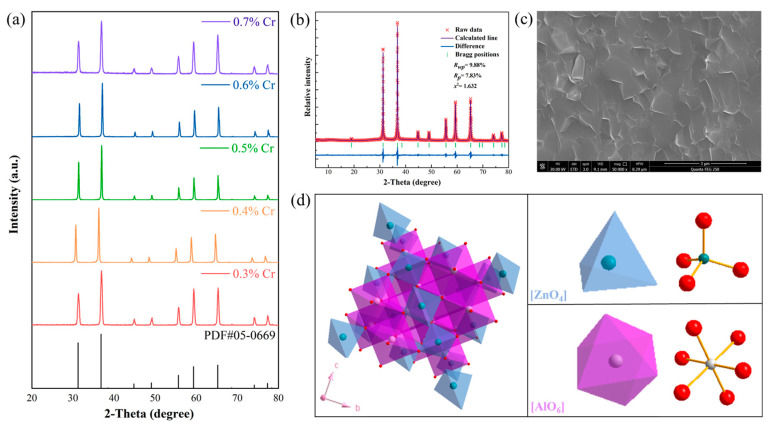
(**a**) XRD patterns of ZAO: xCr^3+^ (x = 0.3%, 0.4%, 0.5%, 0.6%, and 0.7%) phosphor ceramics. (**b**,**c**) XRD refined patterns and cross-sectional SEM images of the ZAO: 0.5%Cr^3+^ sample. (**d**) Spinel crystal structure of ZAO.

Through XRD and its refinement (Figure 1a,b), we conducted phase analysis on ZAO: xCr^3+^ (x = 0.3%, 0.4%, 0.5%, 0.6%, and 0.7%) samples. The XRD diffraction peaks of all samples are clear and consistent, with no apparent impurities or shifts. For ZAO:0.5%Cr^3+^, the refined XRD results show an *R_wp_* of 9.88% and *R_p_* of 7.83%, indicating a good data fit. The detailed refinement parameters are listed in Table 1. According to the refinement results, after doping with 0.5%Cr^3+^, the unit cell parameters and cell volume significantly increase. The doping of Cr^3+^ into the [AlO_6_] octahedra results in an increase in the Al/Cr-O bond length from 1.9117 Å to 2.0161 Å. Simultaneously, the [ZnO_4_] tetrahedra experience a slight contraction, leading to a shorter Zn-O bond. Figure 1d illustrates the schematic diagram of the crystal structure of ZAO, depicting the [ZnO_4_] tetrahedra and [AlO_6_] octahedra. According to the refined XRD and ion coordination information (RAl^3+^ = 0.53 Å, CN = 6; RCr^3+^ = 0.63 Å, CN = 6), Cr^3+^ tends to preferentially occupy the [AlO_6_] octahedra, resulting in the emission of deep red light.

Additionally, we scanned the cross-section of ZAO: 0.5%Cr^3+^ phosphor ceramic using SEM, as shown in Figure 1c, and observed no obvious pores, presenting an overall smooth and dense structure. The successful incorporation of Cr^3+^ into the ZAO matrix was achieved through SPS, with Cr^3+^ effectively integrated into the ZAO lattice.

**Figure 2:**
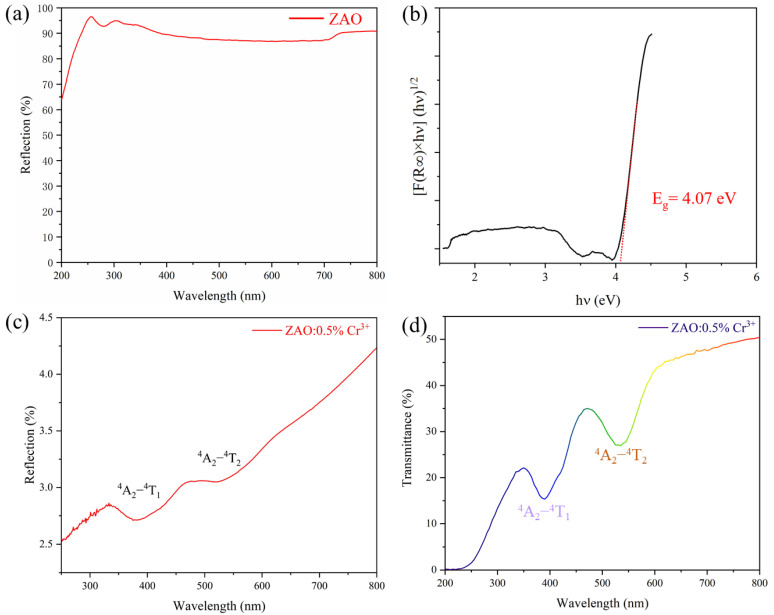
(**a**) DRS and optical bandgap of ZAO matrix (**b**). (**c**,**d**) DRS and transparency of ZAO: 0.5%Cr^3+^ phosphor ceramic.

The optical bandgap determines the intrinsic characteristics of luminescent materials. We conducted diffuse reflectance tests on two samples, ZAO and ZAO: 0.5%Cr^3+^, as shown in Figure 2a,c light, respectively. The ZAO matrix exhibits no apparent absorption peak, and its optical bandgap can be fitted using Formulas (1) and (2) [30]:

(1) $${\left[F\left(R\infty \right)hv\right]}^{n}=A\left(hv-E_{g}\right)$$

(2) $$F\left(R\infty \right)=K/S=(1-R\infty )/2R\infty$$

In the provided expression, *R∞* is the reflectance ratio, *hν* is the photon energy, *K* is the absorption coefficient, *S* is the scattering coefficient, *A* is the proportionality coefficient, and where *n* is 2, it represents the indirect bandgap. As illustrated in Figure 2b, it yields a value of 4.07 eV, suitable for Cr^3+^ doping luminescence [31].

In Figure 2c, two distinct absorption peaks are observed in ZAO: 0.5%Cr^3+^, corresponding to the ^4^A_2_ − ^4^T_1_ transition near 400 nm and the ^4^A_2_ − ^4^T_2_ transition around 535 nm for Cr^3+^ [32]. At the same time, the absence of a distinct absorption peak for Cr^4+^ indicates the highly reducing atmosphere of the SPS, ensuring that the 687 nm deep red emission originates from Cr^3+^. Furthermore, we tested the transparency of ZAO: 0.5%Cr^3+^ ceramic in the visible light region, as depicted in Figure 2d. The maximum transmittance can reach up to 50%, accompanied by two prominent characteristic absorption peaks of Cr^3+^. This pattern is consistent with the diffuse reflectance spectra, indicating that the semi-transparent ceramic allows the transmission of 450 nm blue light and Ce^3+^: YAG yellow light, compensating for the lack of red light.

**Figure 3:**
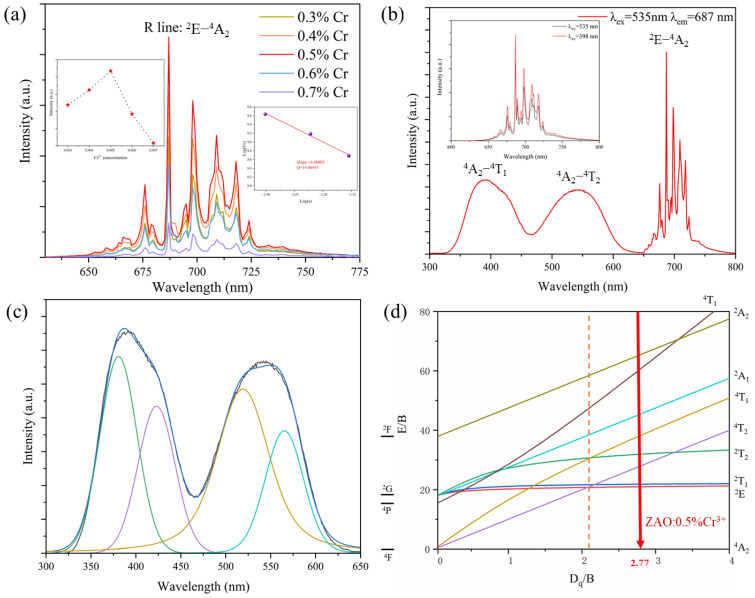
(**a**) PL spectra of ZAO: xCr^3+^ (x = 0.3%, 0.4%, 0.5%, 0.6%, and 0.7%) samples. The left inset shows the relationship between Cr^3+^ concentration and PL intensity, while the right inset displays the fitting plot of log(I/x) and log(x). (**b**,**c**) PLE, PL spectra, and Gaussian fitting plots of the PLE spectrum of ZAO: 0.5%Cr^3+^ sample. (**d**) T-S energy level diagram of the crystal field for Cr^3+^.

In order to find the optimal Cr^3+^ doping concentration, we tested the PL spectra of ZAO: xCr^3+^ (x = 0.3%, 0.4%, 0.5%, 0.6%, and 0.7%) samples, as shown in Figure 3a. ZAO: 0.5%Cr^3+^ exhibited the strongest PL intensity, as illustrated in the inset on the left side of Figure 3a. Hence, we delved into the concentration quenching mechanism, assessing it by calculating the critical distance (*Rc*) through the use of Formula (3) to identify the type of interaction [33]:

(3) $$R_{c}\approx 2{\left(\frac{3V}{4\pi x_{c}N}\right)}^{1/3}$$

The critical concentration, denoted as *X_c_*, is defined as 0.005. Here, *N* is the number of cations per unit cell, specifically 8. The unit cell volume, measured at 528.45 Å^3^, and the calculated critical distance, *Rc*, between two adjacent Cr^3^⁺ ions is determined to be 18.4799 Å. Consequently, the observed concentration quenching mechanism in ZAO:0.5%Cr^3^⁺ is attributed to a multipole interaction, specifically calculated using Dexter theory with Equation (4). [34,35].

(4) $$\frac{I}{x}=\frac{K}{1+{\beta (x)}^{Q/3}}$$

The relationship between the Cr^3^⁺ concentration (*x*), photoluminescence intensity (*I*), and the constants *K* and *β* is expressed in Equation (4). With *Q* taking values of 6, 8, and 10, a linear fitting relationship between *log(I/x)* and *log(x)* was established. The obtained linear fit is illustrated in the right inset of Figure 3a. The slope of this linear fit is calculated as −6.48805, which leads to the determination of *Q* as 119.46415, close to 10. Therefore, the concentration quenching mechanism in ZAO: 0.5%Cr^3+^ phosphor ceramic involves four-quadrupole interaction.

In addition to fitting analysis of the concentration quenching mechanism in ZAO: 0.5%Cr^3+^, we also calculated the crystal field strength of Cr^3+^ in the ZAO matrix to further analyze its PL characteristics. By performing Gaussian fitting on the excitation spectrum of the ZAO: 0.5%Cr^3+^ sample (Figure 3c) and utilizing Formulas (5)–(7) [36]:

(5) $$\frac{D_{q}}{B}=\frac{15(x-8)}{x^{2}-10x}=2.77$$

(6) Dq=E(A24−T24)10

(7) x=E(A24−T14)−E(A24−T24)Dq

where E(^4^A_2_ − ^4^T_2_) is the ^4^A_2_ − ^4^T_2_ transition energy, E(^4^A_2_ − ^4^T_1_) is the ^4^A_2_ − ^4^T_1_ transition energy. The crystal field strength of Cr^3+^ in this sample was calculated to be 2.77, as shown in Figure 3d. This indicates that Cr^3+^ occupies the [AlO_6_] octahedra, experiencing a strong crystal field associated with narrow-band emission. Simultaneously, the dominance of the ^2^E − ^4^A_2_ transition in the emission spectrum suggests its prominent role. 

Figure 3b illustrates the excitation−emission spectra of ZAO: 0.5%Cr^3+^ phosphor ceramic, showcasing a predominant emission peak group centered at 687 nm when excited at 535 nm. We also tested the emission spectra generated by the two main excitation peaks at 398 nm and 535 nm, as shown in the Figure 3b insert. We found no significant difference. To better match the emission peak of YAG:Ce at 535 nm, we chose 535 nm as the excitation light for ZAO:Cr. In the PL spectrum, the 687 nm emission peak corresponds to the ^2^E − ^4^A_2_ transition of Cr^3+^ (Cr^3+^’s zero-phonon R line), while the remaining peaks represent phonon sidebands of the R line. Among them, the 667 nm, 675 nm, and 680 nm peaks represent anti-Stokes shifts, while the 709–724 nm range corresponds to Stokes shifts. 695 nm and 699 nm are the N1 and N2 lines, respectively, resulting from antisite defects in the [AlO_6_] octahedra and [ZnO_4_] tetrahedra [37,38]. To maintain charge balance for the reverse defect-induced Al^3+^ and Zn^2+^ ions, we introduced 1%wt of LiF during the preparation process. This not only ensures charge balance but also facilitates the sintering of the ceramic. Notably, the excitation peaks around 400 nm and 535 nm closely align with the sample’s reflection and transmission absorption peaks, respectively. These peaks correspond to the ^4^A_2_ − ^4^T_1_ and ^4^A_2_ − ^4^T_2_ transitions. The 535 nm excitation band correlates with the emission peak of Ce^3+^: YAG. 

**Figure 4:**
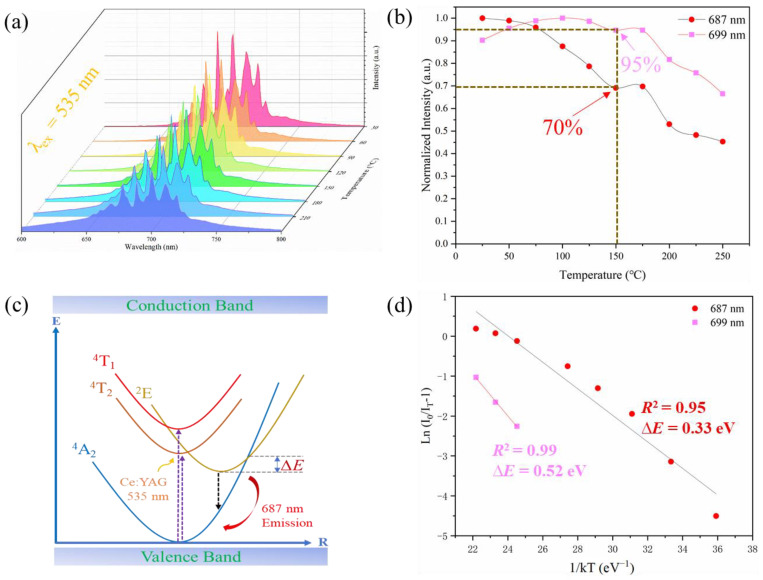
(**a**) Temperature-dependent (30–240 °C) emission spectra of ZAO: 0.5%Cr^3+^ phosphor ceramic excited at 535 nm. (**b**) Temperature variation curves of the emission peaks at 687 nm and 699 nm. (**c**) The configuration coordinate diagram. (**d**) Demonstrates the activation energy fitting.

Beyond phase and spectral analysis, we conducted tests to evaluate the luminescent thermal stability of the phosphor ceramic within the temperature range of 30–240 °C. Temperature-dependent spectra were meticulously recorded, and the results were presented in a 3D plot (Figure 4a), visually depicting the changes in emission peaks at 687 nm and 699 nm corresponding to varying temperatures (Figure 4b). 

In this context, the emission peak at 687 nm maintains approximately 70% of the room temperature PL intensity at a high temperature of 150 °C, while the emission peak at 699 nm retains around 95%. According to previous research reports, the emission peak at 699 nm corresponds to the antisite defect N2 line [37,39]. Therefore, the outstanding thermal stability in this region may be attributed to luminescent center defects. This can be explained by the thermal quenching model shown in Figure 4c. As the temperature rises, the influence of phonons on the excited electrons intensifies. The energy of the ^2^E excited state is thermally activated, reaching the crossover point between the ^2^E and ^4^A_2_ excited states. Subsequently, nonradiative transitions occur to the bottom of the ^4^A_2_ ground state [40]. Because of the presence of antisite defects at 699 nm, the probability of nonradiative transitions is smaller in this region. The difference between the two can also be compared using the Arrhenius Equation (8) [37], with ∆*E* as a parameter.

(8) $$Ln\left(\frac{I_{0}}{I_{T}}-1\right)=LnA-\frac{∆E}{kT}$$

**Figure 5:**
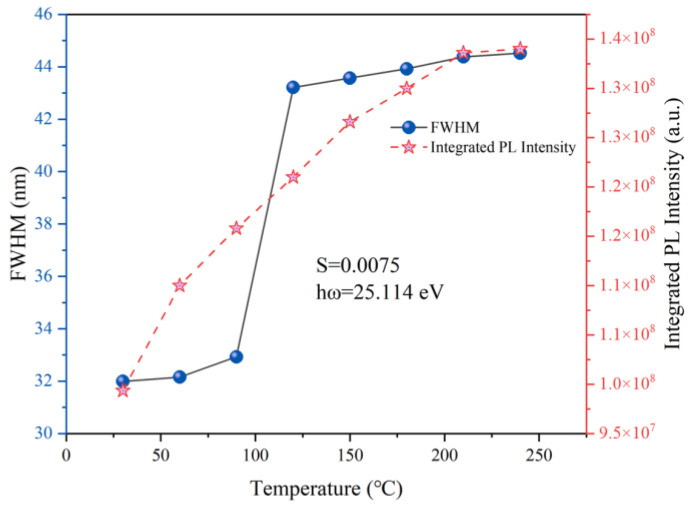
The FWHM, PL integrated intensity, and temperature variation curves for ZnAl_2_O_4_: 0.5%Cr^3+^.

Here, *I_0_* is the initial PL intensity, and *I_T_* is that at temperature *T*. *A* is a constant, and *k* is Boltzmann’s constant (8.6174 × 10^−5^ eV·K^−1^). The linear relationship between *Ln((I*_0_/*I_T_)* − 1) and 1/*kT* is illustrated in Figure 4d. From the fitted data in Figure 4d, the ∆*E* for the emission peak at 687 nm is 0.33 eV, which is significantly lower than the 0.52 eV observed for the emission peak at 699 nm. Therefore, the antisite defects can compensate for the overall PL thermal quenching. To illustrate this, we plotted the temperature dependence of the FWHM and integrated PL intensity for ZAO: 0.5%Cr^3+^ phosphor ceramic, as shown in Figure 5. Due to the existence of the electron-phonon coupling (EPC) effect, the FWHM increases with temperature. Simultaneously, the EPC effect also influences the thermal quenching behavior. We further analyzed the thermal quenching through fitting with the Huang−Rhys formula [1,41].

(9) $$FWHM=2.36\sqrt{S}h\omega \sqrt{coth(\frac{h\omega }{2kT})}$$

In Equation (9), *S* represents the Huang−Rhys factor, indicating the EPC strength; *hν* is the average phonon energy, and *k* is the Boltzmann constant. Through fitting, we obtained an S value of 0.0075. A smaller S value indicates weaker EPC effects, leading to better thermal stability of luminescence, surpassing values reported in most current literature, like Cs_2_NaAlF_6_: Cr^3+^ (S = 4.5), GdAl_3_(BO_3_)_4_: Cr^3+^(S = 5.37), Ca_3_Sc_2_Ge_3_O_12_: Cr^3+^ (S = 4.11) and NaScGe_2_O_6_: Cr^3+^ (S = 1.66) [41,42,43,44]. Additionally, as the FWHM increases and emission from the antisite defects at 699 nm occurs, the integrated PL intensity gradually increases. In conclusion, ZAO: 0.5%Cr^3+^ phosphor ceramic exhibits outstanding luminescence thermal stability. In addition to the fluorescence performance of Cr^3+^ itself, the high thermal conductivity of ZnAl_2_O_4_ spinel also provides positive assistance in thermal quenching.

**Figure 6:**
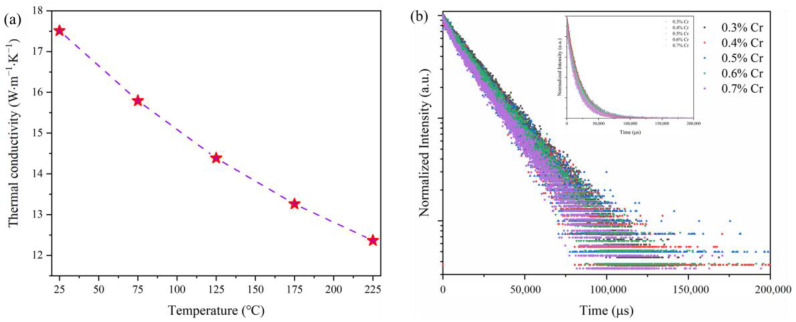
(**a**) Thermal conductivity of ZAO: 0.5%Cr^3+^ phosphor ceramic. (**b**) Fluorescence lifetime spectra at 687 nm for ZAO: xCr^3+^ (x = 0.3%, 0.4%, 0.5%, 0.6%, and 0.7%) samples excited at 535 nm.

Consequently, the thermal conductivity variation curve of ZAO: 0.5%Cr^3+^ phosphor ceramic between 25 °C and 225 °C was evaluated, as shown in Figure 6a. At room temperature, the thermal conductivity of ZAO: 0.5%Cr^3+^ phosphor ceramic is as high as around 17.5 W·m^−1^·K^−1^. Even at a high temperature of 225 °C, it maintains a high thermal conductivity of 12.5 W·m^−1^·K^−1^. Compared to traditional phosphor-mixed transparent silicone gel-encapsulated pc-LEDs, ZAO: 0.5%Cr^3+^ phosphor ceramic can operate continuously in high-temperature environments. The spinel-based phosphor ceramic exhibits excellent heat dissipation performance. Coupled with good luminescent thermal stability, the phosphor ceramic is expected to operate for an extended period in the red-light component of WLED systems (YAG: Ce^3+^ and 450 nm blue LED).

We also evaluated the fluorescence lifetimes of ZAO: xCr^3+^ (x = 0.3%, 0.4%, 0.5%, 0.6%, and 0.7%) samples. Under excitation at 535 nm, the fluorescence lifetime decay at 687 nm is depicted in Figure 6b. The lifetime values obtained through single-exponential fitting are shown in Table 2.

(10) $$I=A+B_{1}\exp\left(-\frac{t}{\tau_{1}}\right)$$

In the given context, *A* and *B*_1_ are constants, and *I* is the PL intensity at time *t*. The variable *τ_1_* corresponds to the decay curve values of the phosphor ceramics. Notably, all samples demonstrate lifetimes in the millisecond range, a trend consistent with prior findings that highlight narrow-band peaks and longer lifetimes (measured in milliseconds) for Cr^3+^ in a strong crystal field [45], aligning with our earlier analysis. As the concentration of Cr^3+^ doping increases, there is a gradual decrease in the values of the decay curves from 20.8 ms to 17.1 ms. This decline is attributed to the heightened occurrence of ion-ion interactions among transition metal ions [46]. Quantum efficiency is also one of the criteria for measuring the practical value of ceramics. We conducted integrating sphere tests for the quantum efficiency of ZAO: 0.5%Cr^3+^ phosphor ceramic, with an internal quantum efficiency (IQE) of 78% and an external quantum efficiency (EQE) of 16%. These quantum efficiency values are among the top in red emitting phosphor ceramics.

**Figure 7:**
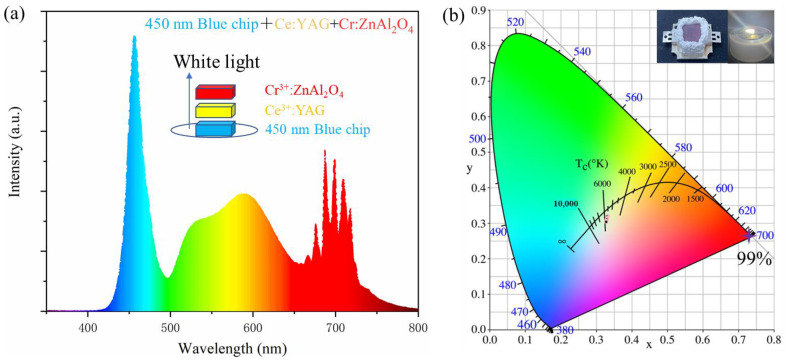
(**a**) PL spectra of ZAO: 0.5%Cr^3+^ compared with Ce^3+^: YAG and 450 nm blue LED encapsulated PL spectra, along with their CIE coordinates (**b**).

Finally, we encapsulated the ZAO: 0.5%Cr^3+^ phosphor ceramic (1 mm thickness) with Ce^3+^: YAG and a 450 nm blue light LED, obtaining its PL spectrum as shown in Figure 7a. The top right corner of Figure 7b shows photos before and after the activation of the white light system. This compensates for the lack of red light. As the driving current increased, the color rendering index R9 increased from 38 to 61.3, attributed to the red emission from Cr^3+^, while the color temperature remained around 5500–5800 K (Figure 7b). Detailed data is presented in Table 3. The white light emitted by the system falls within the white region of the blackbody radiation spectrum, enhancing its practical applicability. The color purity of ZAO: 0.5%Cr^3+^ phosphor ceramic, calculated using Formula (11) [47], can reach 99%. In conclusion, this red phosphor ceramic has the potential to compensate for the lack of red light in white light systems composed of Ce^3+^: YAG and blue light LEDs.

(11) $$\mathrm{Color}\mathrm{purity}=\frac{\sqrt{(x-x_{i}{)}^{2}+(y-y_{i}{)}^{2}}}{\sqrt{(x_{d}-x_{i}{)}^{2}+(y_{d}-y_{i}{)}^{2}}}$$

The coordinates (*x*, *y*), (*x_i_*, *y_i_*), (*x_d_*, *y_d_*) represent the emission color coordinates, white light color coordinates, and dominant wavelength color coordinates of ZAO: 0.5%Cr^3+^ phosphor ceramic, respectively.

## 4. Conclusions

In summary, we successfully prepared semitransparent ZAO: 0.5%Cr^3+^ phosphor ceramic using SPS and analyzed the Cr^3+^ doping sites and spectral properties. Through XRD and refinement, DRS, transmission spectra, crystal field strength, and PLE spectra, we determined that Cr^3+^ occupies [AlO_6_] octahedra, emitting narrow-band red light at 687 nm. The refined data indicates that the doping of Cr^3+^ causes expansion of the [AlO_6_] octahedra and indirect contraction between [ZnO_4_] tetrahedra. Its absorption peak near 535 nm can be effectively excited by Ce^3+^: YAG. The color purity of this phosphor ceramic reaches 99%. Furthermore, ZAO: 0.5%Cr^3+^ phosphor ceramic exhibits a high thermal conductivity of 17.5 W·m^−1^·K^−1^ at a high temperature of 150 °C, and the intensity at 687 nm can be maintained at around 70% of room temperature. The prominent thermal quenching behavior is attributed to the weak EPC effects and the emission from antisite defects at 699 nm. In addition, ZAO: 0.5%Cr^3+^ phosphor ceramic shows a remarkable IQE value, being approximately 78%. When encapsulated with Ce^3+^: YAG for a 450 nm blue LED, it compensates for the lack of red light, adjusts the CT, and improves the R9. This provides valuable insights for the study of WLEDs.

## Figures and Tables

**Table 1:** The detailed refinement parameters of ZAO and ZAO: 0.5%Cr^3+^ samples.

Sample	a (Å)	b (Å)	c (Å)	V (Å^3^)	[Zn-O] (Å)	[Al/Cr-O] (Å)
ZAO	8.0665	8.0665	8.0665	524.87	1.9525	1.9117
ZAO:0.5%Cr^3+^	8.0839	8.0839	8.0839	528.28	1.9475	2.0161

**Table 2:** PL lifetime values of the 687 nm emission peak for ZAO: xCr^3+^ (x = 0.3%, 0.4%, 0.5%, 0.6%, and 0.7%) phosphor ceramics under excitation at 535 nm.

Concentration (%)	0.3	0.4	0.5	0.6	0.7
Lifetime (ms)	20.8	18.9	18.6	18.9	17.1

**Table 3:** Test data for ZAO: 0.5%Cr^3+^ phosphor ceramic, YAG: Ce^3+^ and a 450 nm LED encapsulation.

Drive Current (mA)	Color Coordinates (x, y)	Luminous Efficacy (lm/W)	Luminous Flux (lm)	Color Rendering Index	R9	Color Temperature (K)
* 50	(0.3063, 0.3421)	8.3	3.2	75.7	−10	6735
50	(0.3315, 0.3206)	6.1	2.4	85	38	5543
100	(0.3311, 0.3189)	6.1	4.9	85.3	38.9	5558
300	(0.3304, 0.3147)	5.5	14.3	85.9	41.9	5599
500	(0.3303, 0.3128)	5.0	22.6	86.1	42.9	5606
800	(0.329, 0.3085)	4.4	33.6	86.9	51.1	5683
1000	(0.3283, 0.3062)	4.1	40.1	87.3	55.9	5730
1300	(0.3275, 0.3026)	3.9	50.3	87.8	61.3	5780

* Initial color characteristics of the LED (YAG: Ce^3+^ phosphor ceramic packaged with a 450 nm LED).

## Data Availability

The data of this paper are available on request from the corresponding author.
